# Supplementary material for: Cellular Heterogeneity–Adjusted cLonal Methylation (CHALM) improves prediction of gene expression
Source: Nat Commun. 2021 Jan 15;12:400. doi: 10.1038/s41467-020-20492-7 (PMC7811027; doi:10.1038/s41467-020-20492-7)
Supplement: Supplementary file 3 — Description of Additional Supplementary Files [file 41467_2020_20492_MOESM3_ESM.pdf]

Description of Additional Supplementary Files

Supplementary Data 1: List of promoter CGIs defined in hg19 genome reference

Supplementary Data 2: List of differentially methylated promoter CGIs between lung tumor-normal pair

Supplementary Data 3: List of de novo differentially methylated regions between LUAD and SCLC

Supplementary Data 4: List of datasets used in this study
