# Supplementary material for: Cellular Heterogeneity–Adjusted cLonal Methylation (CHALM) improves prediction of gene expression
Source: Nat Commun. 2021 Jan 15;12:400. doi: 10.1038/s41467-020-20492-7 (PMC7811027; doi:10.1038/s41467-020-20492-7)
Supplement: Supplementary file 8 — Reporting summary [file 41467_2020_20492_MOESM8_ESM.pdf]

nature research

Corresponding author(s): Wei Li

Last updated by author(s): Sep 7, 2020

Reporting Summary

Nature Research wishes to improve the reproducibility of the work that we publish. This form provides structure for consistency and transparency in reporting. For further information on Nature Research policies, see our Editorial Policies and the Editorial Policy Checklist.

Statistics

For all statistical analyses, confirm that the following items are present in the figure legend, table legend, main text, or Methods section.

n/a	Confirmed	
☐	☒	The exact sample size (*n*) for each experimental group/condition, given as a discrete number and unit of measurement
☒	☐	A statement on whether measurements were taken from distinct samples or whether the same sample was measured repeatedly
☐	☒	The statistical test(s) used AND whether they are one- or two-sided<br>*Only common tests should be described solely by name; describe more complex techniques in the Methods section.*
☒	☐	A description of all covariates tested
☐	☒	A description of any assumptions or corrections, such as tests of normality and adjustment for multiple comparisons
☐	☒	A full description of the statistical parameters including central tendency (e.g. means) or other basic estimates (e.g. regression coefficient) AND variation (e.g. standard deviation) or associated estimates of uncertainty (e.g. confidence intervals)
☐	☒	For null hypothesis testing, the test statistic (e.g. *F*, *t*, *r*) with confidence intervals, effect sizes, degrees of freedom and *P* value noted<br>*Give P values as exact values whenever suitable.*
☒	☐	For Bayesian analysis, information on the choice of priors and Markov chain Monte Carlo settings
☒	☐	For hierarchical and complex designs, identification of the appropriate level for tests and full reporting of outcomes
☒	☐	Estimates of effect sizes (e.g. Cohen's *d*, Pearson's *r*), indicating how they were calculated

Our web collection on statistics for biologists contains articles on many of the points above.

Software and code

Policy information about availability of computer code

Data collection	RNA-seq process: Trimmomatic (0.35); TopHat (2.1.0); Cufflinks (2.2.1); HTSeq (2.7); DEseq2 (1.20)<br>DNA methylation data analysis: BSMAP (2.90); Metilene (0.2-7); DAVID (6.8); Enrichr (online tool)<br>ChIP-seq data analysis: Bowtie2 (2.2.7); MACS (2.1.0)<br>R packages: bcv (1.0.1)<br>Python packages: pytorch (1.2)
Data analysis	Analysis related to CHALM calculation and gene expression prediction by deep learning are conducted with custom developed tools, which are available at: https://github.com/JianfengXu93/CHALM;<br>DMR calling tool: Metilene (0.2-7);<br>Pathway enrichment analysis are conducted with DAVID (6.8) and Enrichr (online tool);<br>All other custom codes related to this work are available upon request.

For manuscripts utilizing custom algorithms or software that are central to the research but not yet described in published literature, software must be made available to editors and reviewers. We strongly encourage code deposition in a community repository (e.g. GitHub). See the Nature Research guidelines for submitting code & software for further information.

Data

Policy information about availability of data

All manuscripts must include a data availability statement. This statement should provide the following information, where applicable:

- Accession codes, unique identifiers, or web links for publicly available datasets
- A list of figures that have associated raw data
- A description of any restrictions on data availability

Public datasets from GEO and Roadmap project are used in this study. Accession number and links to all related datasets can be found in the method section. Processed DNA methylated data (differentially methylated CGIs or de novo DMRs) are provided in supplementary datasets. Other processed data will be provided upon request.

Field-specific reporting

Please select the one below that is the best fit for your research. If you are not sure, read the appropriate sections before making your selection.

- [x] Life sciences
- [ ] Behavioural & social sciences
- [ ] Ecological, evolutionary & environmental sciences

For a reference copy of the document with all sections, see nature.com/documents/nr-reporting-summary-flat.pdf

Life sciences study design

All studies must disclose on these points even when the disclosure is negative.

Sample size	Since only public datasets are used for this work, we did not perform sample size calculations. The sample sizes is determined by the data availability.
Data exclusions	For the RNA-seq data of TCGA-LUAD, only 79 samples are randomly selected to matched the sample size of the SCLC RNA-seq dataset (GSE60052).
Replication	All results have been repeated for at least two times in different datasets if multiple datasets are available. Results are consistent between repeated analyses.
Randomization	Samples are selected randomly if the whole dataset is not used.
Blinding	Since only public datasets are used for this work, this is not applicable.

Reporting for specific materials, systems and methods

We require information from authors about some types of materials, experimental systems and methods used in many studies. Here, indicate whether each material, system or method listed is relevant to your study. If you are not sure if a list item applies to your research, read the appropriate section before selecting a response.

Materials & experimental systems

n/a	Involved in the study	
x		Antibodies
x		Eukaryotic cell lines
x		Palaeontology and archaeology
x		Animals and other organisms
x		Human research participants
x		Clinical data
x		Dual use research of concern

Methods

n/a	Involved in the study	
x		ChIP-seq
x		Flow cytometry
x		MRI-based neuroimaging
